# Efficacy of Intra-Articular Injection of Celecoxib in a Rabbit Model of Osteoarthritis

**DOI:** 10.3390/ijms11104106

**Published:** 2010-10-21

**Authors:** Dinghua Jiang, Jun Zou, Lixin Huang, Qin Shi, Xuesong Zhu, Genlin Wang, Huilin Yang

**Affiliations:** Department of Orthopaedic Surgery, The First Affiliated Hospital of Soochow University, 188 Shizi St., Suzhou, Jiangsu 215006, China; E-Mails: zoujun105@126.com (D.H.J.); jzou@suda.edu.cn (J.Z.); qshisz@gmail.com (Q.S.); zhuxs@126.com (X.S.Z.); wglpaper@126.com (G.L.W.); hlyang@suda.edu.cn (H.L.Y.)

**Keywords:** celecoxib, hyaluronic acid, intra-articular injection, osteoarthritis

## Abstract

Introduction: Osteoarthritis is the most common form of arthritis. It is a slowly progressive joint disease typically seen in middle-age to elderly people. Intra-articular injection of hyaluronic acid is a well-documented treatment for knee osteoarthritis. Celebrex^®^ (celecoxib) is a novel nonsteroidal anti-inflammatory drug, which could help to reduce inflammation and to reduce pain. The aim of this study was to evaluate the effects of intra-articular injection of celecoxib in a rabbit osteoarthritis model. Methods: Thirty New Zealand white rabbits underwent unilateral knee joint surgery using the Hulth technique. Six weeks post-surgery, the animals were randomly divided into three groups, and each group was respectively given weekly intra-articular injections with Celebrex^®^, hyaluronic acid and saline. On the sixth week, the results were assessed in rabbit models by gross observation, histological evaluation, and expression of IL-1β, TNF-α, MMP-3. Results: In the group given Celebrex^®^ and hyaluronic acid, the pathological changes in the rabbit articular cartilage improved significantly, much more than in the saline group. The statistically significant suppression of IL-1β, TNF-α, MMP-3 was shown in the Celebrex group. No significant differences were detected between two treatment groups. Conclusions: Intra-articular injection of celecoxib is beneficial for knee osteoarthritis. It might repair and protect early osteoarthritis cartilage by delaying cartilage degeneration and impairing the function of inflammatory mediators, therefore, intra-articular injection of celecoxib can be used as an alternative to the current treatment of osteoarthritis.

## 1. Introduction

Osteoarthritis (OA) is a degenerative joint disease occurring mostly occurs in the knee and commonly seen in middle-aged and elderly adults [1]. Knee OA is a degenerative disease that manifests as knee joint dysfunction that results from joint destruction and deformation. It’s caused by primary and secondary degeneration and structural disturbance of knee joint cartilage, and is accompanied by subchondral bone hyperplasia and cartilage denudation. The pain and disability due to OA significantly affects patients’ quality of life. Therefore, the importance and impact of OA have recently been compared to the threat of cardiovascular attack [2].

The treatment for early OA mainly consists of relieving its clinical symptoms, delaying joint degeneration. Non-surgical modalities for OA management include weight loss, exercise, activity modification, assistive devices, non-steroidal anti-inflammatory drugs (NSAIDs), analgesics, and intra-articular hyaluronic acid (HA) preparations [3]. Intra-articular treatment currently represents the standard method for affecting joint pain and inflammation in patients with arthritides and so-called activated osteoarthritis accompanied by inflammatory processes [4]. Intra-articular treatment is effective in relieving pain and prolonging degeneration, therefore it has been one of the main treatments for osteoarthritis [5,6]. Celebrex^®^ (celecoxib, Pfizer Inc., NY, USA) is a type of NASID and a novel selective COX-2 inhibitor, which can selectively suppress inflammation relevant COX-2, while preserving the physical functions of COX-1. This drug avoids gastrointestinal complications caused by traditional NSAIDs. In this study, we attempted to determine whether intra-articular injection of celecoxib would have the anti-inflammatory advantages, while also maintaining its protective effect on articular cartilage, as well as HA, in a rabbit OA model.

## 2. Materials and Methods

### 2.1. Study Design

Thirty New Zealand white rabbits (irrespective of sex), each weighing 2.2–3.0 Kg, were provided by the Experimental Animal Center of University. Under anesthesia with 10% chloral hydrate (4 mL/kg body weight), OA models were established by performing unilateral knee joint surgery as described by Hulth [7]. Through a medial parapatellar incision, knee joints were opened, and anterior and posterior cruciate ligaments were transected and medial meniscus excised. Postoperatively, all animals were permitted free cage activity. Six weeks after the surgery, the animals were divided into three groups (n = 10 in each group): celecoxib group; HA group and control group. For animals in the treatment groups, 0.3 mL of 0.4% celecoxib, or 1% HA (Baker Norton Co., Kunming, China) was injected weekly into the affected joint for five weeks, while 0.3 mL of normal saline was administered to those in the control group.

### 2.2. Gross Pathology Observation and Histological Evaluation

After five weeks, the rabbits were sacrificed and femoral condyles were collected for observation. After macroscopic examination, isolated samples were fixed with 10% neutral buffered formalin and decalcified with 20% EDTA solution for further histological evaluation. The decalcified samples were embedded in paraffin, and standard frontal sections of 5 μm sections were prepared and stained with hematoxylin-eosin in the lateral part of the femoral condyle cartilage according to gross observation. The weight bearing area of the femoral condyle was delimited and cartilage degradation was assessed using the Mankin’s scoring system [8]. All sections were graded by three independent observers that were kept unaware of the treatment groups.

### 2.3. Measurement of IL-1β and TNF-α in Synovial Fluid

At the five-week time point, synovial fluid was collected from the operated knee before sacrifice. The samples were stored at −30°C until assayed. The contents of IL-1β and TNF-α in synovial fluid were measured by ELISA kits (Senxiong Co., Shanghai, China).

### 2.4. Measurement of mRNA Expression of MMP-3

After sacrifice, synoviums of the operated knee were harvested for measurement of mRNA expression of MMP-3. Primers were designed and synthesized. Total RNA was extracted, measured, reverse transcripted, and amplified by reverse transcription-polymerase chain reaction (RT-PCR) (Perkin-Elmer, Norwalk, USA). The PCR products were separated on 1.5% agarose gels and visualized by ethidium bromide staining. Ethidium bromide-stained gels were digitally photographed. The relative levels of mRNAs were normalized to the mean gray value of β-actin and then expressed as relative mRNA value for each specimen.

### 2.5. Statistical Analysis

The data was processed by the SPSS11.5 software package and expressed as *x̄* ± s. Differences between groups were compared by t-test and considered to be statistically significant if *P* < 0.05.

## 3. Results

### 3.1. Gross Pathological Observation

**Figure 1:**
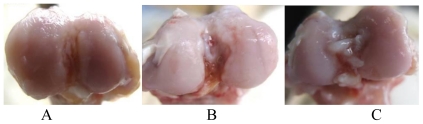
Gross pathological observation of the femoral condyle in saline (**A**), celecoxib (**B**) and HA (**C**) groups.

Normal rabbit knee articular cartilage is smooth and lustrous with a light blue-white color. In the control group, cartilage appeared to be yellowish-white, lackluster and its appearance displayed significant cracks and cartilage defects; osteophyte-like structures were observed in the femoral condyle and both sides of the tibial plateau (Figure 1A). In the treatment groups, both the celecoxib group and HA group, cartilage lesions associated with OA were clearly seen, but the severity was milder than in the control group (Figure 1B, C).

### 3.2. Histological Evaluation

**Figure 2:**
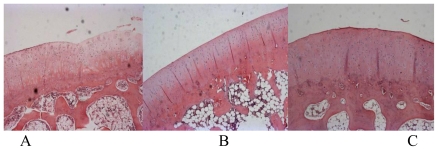
Histological findings of the femoral condyles in saline (**A**); celecoxib (**B**) and HA (**C**) groups.

**Figure 3:**
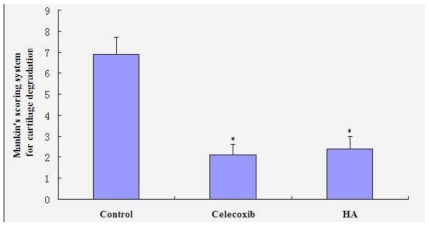
Histological scores of cartilage according to Mankin scoring system. * P < 0.05 compared to saline group.

In the control group, findings included degenerative/necrotic and disarranged chondrocytes, significant ulcerous lesions in the deep cartilage layer, considerable inflammatory exudates, newly formed capillaries and fibroblast proliferation, fibroplasia in the bottom of the ulcers, and degenerative deep chondrocytes (Figure 2A). In both two treatment groups, observations included degenerative/necrotic or shed perichondrium, degenerative/necrotic and disarranged superficial chondrocytes, and a small amount of inflammatory exudates, erosion, and sparse capillary and fibroblast proliferation (Figure 2B, C). Mankin’s scores in the celecoxib (2.1 ± 0.5) and HA (2.4 ± 0.6) groups were significantly lower than in the control group (6.9 ± 0.8) (P < 0.05), as seen in Figure 3. However, no significant differences between the two treatment groups were observed.

### 3.3. IL-1β and TNF-α in Synovial Fluid

**Figure 4:**
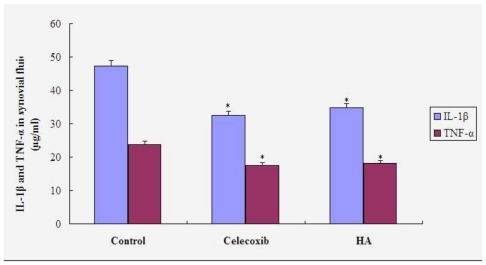
Contents of IL-1β and TNF-α in synovial fluid. * P < 0.05 compared to saline group.

Figure 4 shows the results of the measurements of IL-1β and TNF-α in synovial fluid from each group. Differences in both IL-1β and TNF-α contents were significant between treatment and control groups (P < 0.05), but not significant between the three treatment groups (P > 0.05).

### 3.4. mRNA Expression of MMP-3

**Figure 5:**
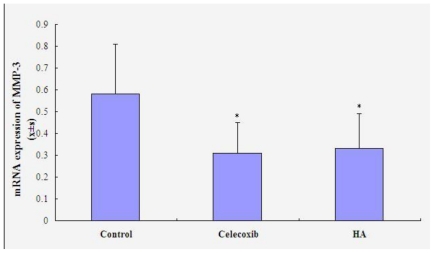
mRNA expression of MMP-3 in synovium. * P < 0.05 compared to saline group.

As shown in Figure 5, there was a statistically significant decrease in mRNA expression of MMP-3 in the celecoxib group (P < 0.05), comparing to control group (0.58 ± 0.23). However, it did not significantly differ between the two treatment groups (celecoxib 0.31 ± 0.14, HA 0.33 ± 0.16) (P > 0.05).

## 4. Discussion

The exact pathogenesis of OA remains unclear so far. Generally, it is an articular cartilage injury caused by a coupling imbalance in the degradation and synthesis of chondrocytes, extracellular matrix and subchondral bone. This is due to a biomechanical disorder induced by interactions between various biological factors and mechanical injury factors. Many studies have confirmed that several biological factors are involved in the development of OA, including IL-1β, TNF-α, TGF-β, MMPs and NO. IL-1β and TNF-α are found to be important regulators of inflammatory reaction [9,10].

IL-1β is one of the most important cytokines responsible for destruction of articular cartilage. It functions by: (1) mediating the most effective destruction of cartilage; (2) regulating bone re-absorption; (3) enhancing the destructive inflammatory process of OA by stimulating the production of IL-8 in synovial tissue macrophages, fibroblasts and chondrocytes; (4) promoting scarring and fibrosis by directly or indirectly stimulating fibroblast proliferation, and (5) prolonging the survival of neutrophils and boosting the inflammatory process. TNF-α is only 3% homologous with IL-1β. The biological activity of TNF-α is 100 times lower than that of IL-1β, which cannot activate the murine T cell effect. TNF-α may induce the production of other cytokines including IL-1β and monocyte/granulocyte stimulating factor, while IL-1β may increase the activity of TNF-α. There is a synergistic effect between TNF-α and IL-1β that may mediate destruction of joint tissues [11]. In this study, we found that the content of TNF-α and IL-1β was significantly suppressed in the celecoxib group in comparison to that in the control group injected with saline. The results suggest that celecoxib may suppress the inflammatory response in inflamed joints by inhibiting the expression of TNF-α and IL-1β.

HA is a main component in synovial fluid and cartilage matrix. HA in synovial fluid is primarily produced by type B synovial lining cells. A normal concentration of normal HA in synovial fluid is critical for the maintenance of joint function [12]. Intra-articular treatment with HA for OA knee pain is widely accepted [13]. This modality not only replenishes the intra-articular HA, but also inhibits inflammatory mediators’ synthesis, relieves joint pain, postpones the development osteoarthritis [14,15]. Our study indicated that celecoxib injection might repair and protect against early OA cartilage by delaying cartilage degeneration via inhibiting MMP-3 expression as well as HA group. It was also showed in our macroscopic and histological analyses. Since MMP-3 has the potent ability to degrade the proteoglycan of cartilage, MMP-3 is recognized as an enzyme that plays a part in the destruction of cartilage and bone in OA [16]. The results of this study demonstrated that the level of MMP-3 was elevated and sustained in the control group treated by injecting saline, whereas elevation of MMP-3 levels was significantly suppressed in the celecoxib and HA groups. However, there is not a significant difference in these two treatment groups. Therefore, it is expected that both of celecoxib and HA would be beneficial in the prevention of cartilage damage, as reflected by a reduction in MMP-3 levels.

## 5. Conclusions

In summary, our results suggest that intra-articular injection of celecoxib is an effective therapeutic method in an OA model. Celecoxib might not only provide substantially benefits to OA by suppressing of the levels of TNF-α and IL-1, but also maintain cellular activity and extracellular matrix synthesis by reducing MMP-3 synthesis. It could protect the cartilage matrix from degradation with correspondingly improved efficiency observed in the macroscopic and histological analyses. This novel therapeutic strategy could be promising treatment option for OA.
